# Delivery of the HIV Service and Telemedicine Through Effective Patient-Reported Outcomes (+STEP) Intervention to Increase Screening and Treatment of Mental Health and Substance Use Disorders for People Living With HIV in Alabama: Protocol for an Effectiveness-Implementation Study

**DOI:** 10.2196/40470

**Published:** 2023-08-15

**Authors:** Kelly W Gagnon, Stefan Baral, Dustin Long, Alfredo L Guzman, Bernadette Johnson, Greer Burkholder, James Willig, Michael Mugavero, Margaret Baldwin, Susanne Fogger, Thomas Creger, Karen Cropsey, Ellen Eaton

**Affiliations:** 1 Division of Infectious Disease Heersink School of Medicine University of Alabama at Birmingham Birmingham, AL United States; 2 Center for Addiction and Pain Prevention and Intervention University of Alabama at Birmingham Birmingham, AL United States; 3 Department of Epidemiology Bloomberg School of Public Health Johns Hopkins University Baltimore, MD United States; 4 Centers for AIDS Research Heersink School of Medicine University of Alabama at Birmingham Birmingham, AL United States; 5 Department of Biostatistics School of Public Health University of Alabama at Birmingham Birmingham, AL United States; 6 Nursing Family, Community and Health Systems School of Nursing University of Alabama at Birmingham Birmingham, AL United States; 7 Department of Psychiatry and Behavioral Neurobiology Heersink School of Medicine University of Alabama at Birmingham Birmingham, AL United States

**Keywords:** clinics, diagnosis, engagement, HIV, implementation science, intervention, interview, mental health, patient reported outcomes, protocol, screening, substance use, telemedicine, treatment, USA, viral

## Abstract

**Background:**

The syndemic of mental health (MH) and substance use disorders (SUDs) is common among persons living with HIV and jeopardizes HIV treatment adherence, engagement in care, and viral load suppression. Electronic patient-reported outcomes (ePROs), completed through tablet or computer, and telemedicine are evidence- and technology-based interventions that have been used to successfully increase screening and treatment, respectively, a model that holds promise for persons living with HIV. To date, there is limited guidance on implementing ePROs and telemedicine into HIV clinical practice even though it is well known that these evidence-based tools improve diagnosis and access to care.

**Objective:**

To address this, we aim to conduct a multicomponent intervention for persons living with HIV, including the delivery of HIV services and telemedicine through effective ePROs (+STEP), to increase screening and treatment of MH and SUD in Ryan White HIV/AIDS Program (RWHAP)–funded clinics in Alabama.

**Methods:**

Through this intervention, we will conduct a readiness, acceptability, and accessibility assessment and implement +STEP to improve the diagnosis and treatment of MH and SUD at RWHAP clinics in Alabama. To describe implementation strategies that address barriers to the uptake of +STEP in RWHAP clinics, we will conduct qualitative interviews in years 1 (early implementation), 2 (scale up), and 4 (maintenance) with patients and key staff to evaluate barriers, facilitators, and implementation strategies. Our Results will enable us to modify strategies to enhance +STEP penetration over time and inform the implementation blueprint, which we will develop for both RWHAP clinics in Alabama and future sites. We will assess the impact of implementing +STEP on diagnoses, referrals, and health care use related to MH, SUD, and HIV by comparing clinical outcomes from patients receiving these interventions (ePROs and telemedicine) with historical controls.

**Results:**

The first study site began implementation in April 2022. A total of 2 additional sites have initiated ePROs. Final results are expected in 2026. The results of this study will provide a foundation for future research expanding access to ePROs for improved diagnosis linked to telemedicine access to accelerate patients along the continuum of care from MH and SUD diagnosis to treatment.

**Conclusions:**

Achieving the end of the HIV epidemic in the United States necessitates programs that accelerate movement across the MH and SUD care continuum from diagnosis to treatment for persons living with HIV. Scaling these services represents a path toward improved treatment outcomes with both individual health and population-level prevention benefits of sustained HIV viral suppression in the era of undetectable=untransmittable (U=U). This study will address this evidence gap through the evaluation of the implementation of +STEP to establish the necessary systems and processes to screen, identify, and better treat substance use and MH for people living with HIV.

**International Registered Report Identifier (IRRID):**

DERR1-10.2196/40470

## Introduction

Almost 10 million people in the United States are estimated to have both substance use and mental health (MH) disorders [1]. In Alabama, the burden of MH is higher than the national average with nearly 1 in 4 people meeting criteria for any depressive disorder compared to the national average of under 20% [2]. Additionally, approximately 8% of Alabama residents are diagnosed with a substance use disorder (SUD) each year [3]. The syndemic of MH and SUDs is common among persons living with HIV, with estimated prevalence rates of 39% and 48%, respectively [4,5]. This syndemic jeopardizes HIV treatment adherence, engagement in care, and viral load (VL) suppression [6]. Specifically, MH and SUD are associated with reduced antiretroviral treatment (ART) adherence, high viral load levels, and increased mortality [7,8]. persons living with HIV with MH and SUD may be reluctant to report these conditions due to stigma, medical mistrust, or social desirability bias, but diagnosis is an essential first step toward management of these comorbidities [6,9]. Following diagnosis, improving access to MH and SUD treatment has the potential to reduce HIV transmission risks by fostering sustained viral suppression.

Treating MH conditions and reducing drug use improves ART adherence and VL suppression [10-12]. Thus, MH and SUD should be monitored often and addressed rapidly, a process facilitated by real-time electronic patient reported outcome (ePRO) data. A recent Cochrane review of 116 randomized control trials demonstrated that ePROs are a successful mechanism to improve patient-provider communication, diagnosis, and disease control [13]. While there is currently insufficient evidence that ePROs improve distal clinical outcomes, research shows ePROs can improve the process of care by collecting information directly from patients, minimizing potential for judgment and social desirability bias [14-16]. ePROs completed on a laptop or tablet using validated tools, such as the Patient Health Questionnaire-9 (PHQ-9), are replicable and provide longitudinal monitoring of the overall health of persons living with HIV [6,17-19]. This evidence-based screening mechanism enables clinics to respond with individualized counseling and education, allowing resource-limited clinics to prioritize scarce resources to those with active psychosocial barriers that may impair ART adherence and VL suppression.

Alabama is 1 of 7 states with the greatest rural HIV burden nationally, and the rural, resource-limited region poses additional barriers in treating psychosocial comorbidities in persons living with HIV. Challenges include a lack of providers, especially MH and addiction treatment providers, long commutes across rural settings to clinics, and a lack of public transportation. Lack of Medicaid expansion substantially widens this treatment gap [9,20-22]. Telemedicine offers an opportunity to overcome barriers to treatment in southern and rural states, such as lack of transportation, time away from work and childcare, costs, and stigma [22,23].

There is a need for programs that improve and accelerate the diagnosis and management of MH and SUD among persons living with HIV and promote the individual health and population level prevention benefits of sustained HIV viral suppression in the era of undetectable=untransmittable (U=U). ePROs, completed through tablet or computer, and telemedicine are evidence- and technology-based interventions that have been used to successfully increase screening and treatment, respectively, a model that holds promise for persons living with HIV [24,25]. To date, there has been a lack of consensus in the field on the implementation of ePROs, with specifically limited guidance on how to implement ePROs and telemedicine into HIV clinical practice. A majority of existing implementation recommendations are for integration into clinical practice generally and are not specific to HIV settings [14,26-28]. Additionally, implementation studies to date were not based in the Deep South, where at-risk populations face unique barriers to MH, SUD, and HIV care [14,26-29]. Given that ePROs improve diagnosis and access to care, additional recommendations specific to the integration of ePROs into clinical HIV settings are necessary to improve the implementation and dissemination of this evidence-based tool to address the HIV epidemic [6,30]. To address limited guidance specific to HIV clinical settings, we are conducting a multicomponent intervention for persons living with HIV, delivery of HIV services, and telemedicine through effective ePROs (+STEP), to increase screening and treatment of MH and SUD in Ryan White HIV/AIDS Program (RWHAP)-funded clinics in Alabama. The aims of this study are the following:

- Aim 1: to conduct readiness, acceptability, and accessibility assessments through qualitative interviews and a quantitative survey followed by the implementation of +STEP to improve the diagnosis and treatment of MH and SUD at RWHAP clinics in Alabama.
- Aim 2: to describe implementation strategies that address barriers to uptake of +STEP in RWHAP clinics. We will conduct qualitative interviews in years 1 (early implementation), 2 (scale up), and 4 (maintenance) with patients and key staff to evaluate barriers, facilitators, and implementation strategies. Results will enable us to modify strategies to enhance +STEP penetration over time and inform the implementation blueprint, which we will develop for both RWHAP clinics in Alabama and future sites.
- Aim 3: to measure the impact of implementing +STEP on diagnoses, referrals, and health care use related to MH, SUD, and HIV by comparing clinical outcomes from patients receiving these interventions (ePROs and telemedicine) with historical controls. We will complete a quantitative assessment of clinic-level diagnoses (MH and SUD) and patient-level indicators (screening, reporting, and treatment for MH and SUDs) relative to historical RWHAP reports using segmental regression (previous 3 years).

This will be the first study to evaluate integrated implementation of 2 effective evidence-based technologies—ePROs and telemedicine—in serving people living with HIV in Alabama. The results of this study will provide a foundation for future research expanding access to ePROs for improved diagnosis linked to telemedicine access to accelerate patients along the continuum of care from MH and SUD diagnosis to treatment.

## Methods

### Setting

+STEP is being implemented in RWHAP-funded clinics in Alabama. RWHAP-funded clinics provide medical care, medications, and essential support services to low-income persons living with HIV. In addition to providing essential care to persons living with HIV, these clinics aim to improve HIV-related health outcomes and reduce HIV transmission in their local communities [31]. Of the 9 RWHAP-funded clinics in Alabama, a total of 5 have agreed to participate in the study.

### Study Design

We will conduct a stepped-wedge hybrid implementation-effectiveness type 1-design study, allowing the testing of implementation strategies while simultaneously evaluating the impact of the intervention on clinical outcomes of +STEP. Key staff from the participating sites, including clinic leadership, patient representatives, and clinicians (social workers, nurses), will participate in clinic-level trainings and assessments. Each site will identify key staff and patients who will be invited to join the advisory board. Implementation outcomes will be assessed using Proctor’s Implementation Framework [32].

**Figure 1:**
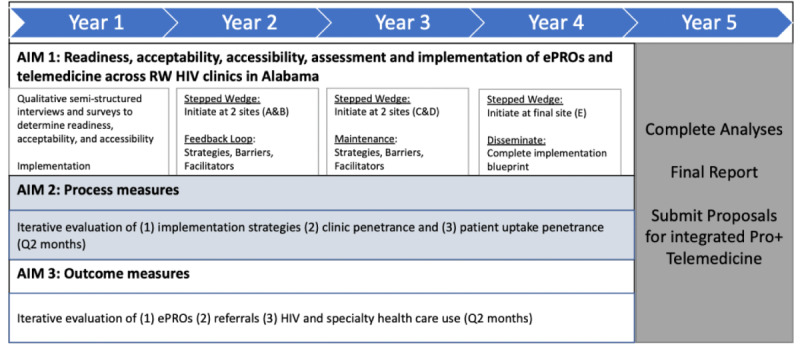
Study timeline of specific aims. ePRO: electronic patient reported outcome; RW: Ryan White; Q: quarter.

For aim 1, we will conduct qualitative, semi-structured interviews and surveys with key staff to assess organizational readiness and existing protocols. Specifically, interviews will explore each clinic’s current resources and protocols, team structure, screening procedures, and barriers to telehealth. Surveys will measure organizational readiness. We will also survey patients with personal MH or SUD experiences to determine the accessibility and acceptability of ePROs and telehealth. We will then implement a multicomponent intervention with ePROs, targeted training to frontline providers, and telemedicine. We will assess fidelity and process measures, including clinic adoption and patient participation, as part of routine care in community-based RWHAP clinics. For aim 2, we will use the Consolidated Framework for Implementation Research (CFIR) to conduct a qualitative evaluation of implementation strategies, barriers, and facilitators to +STEP uptake. [33] Thus, Aim 2 will allow us to develop and refine an implementation blueprint. For Aim 3, we will evaluate the intervention impact on patient and clinic outcomes using patient-reported data (eg, MH, ART adherence), use of services (ie, clinical encounters and telemedicine), and retention in care, a proximal measure of VL suppression. A timeline for implementation of this study can be found in Figure 1.

### Intervention

+STEP will combine 3 components in a novel, multicomponent intervention**:** ePROs, targeted knowledge to frontline providers and staff, and telemedicine services for the management of MH and SUD. The intervention will include ePRO surveys adapted at the clinic level based on the results of the readiness, acceptability, and accessibility assessments. The ePROs will include validated surveys of MH and SUD, and final instruments will be selected following key staff meetings in order to ensure that they meet the needs and preferences of sites. Key staff from the participating sites, including clinic leadership, patient representatives, and clinicians (social workers, nurses), will participate in clinic-level trainings and assessments. The intervention will also include ongoing targeted trainings for frontline staff emphasizing the importance of diagnosis and evidence-based treatment of MH and SUD [30,34]. We will educate participants on the role of stigma and language as barriers to MH and SUD treatment [35]. With input from clinic-level key staff, we will design training on how to respond to ePRO results, including evidence of MH and suicidal ideation, according to clinic-specific policies and procedures. Telemedicine from the University of Alabama at Birmingham (UAB) eMedicine Center allows persons living with HIV to leverage UAB’s telemedicine experience and infrastructure in 55 out of 57 counties across Alabama. Patients referred to telemedicine will have access to a central contact at UAB’s eMedicine Center who will arrange an appointment at the closest referral site.

Implementation strategies: we will identify and train a +STEP champion at each clinical site who will receive additional hands-on training on the +STEP protocols, technology, and troubleshooting [36]. Implementation champions are an established implementation strategy to influence and facilitate change within their organization [37]. +STEP champions will be identified at each site before implementation and join regular meetings with the +STEP research team to discuss ongoing challenges and successes. Additional implementation strategies to improve implementation will be identified and used during the evaluation of +STEP implementation and as additional sites initiate the intervention.

### Study Population

Participants include staff at RWHAP-funded clinics in Alabama and the persons living with HIV receiving care at these clinics. All adult (18 years of age or older) patients will be included in the analysis regardless of race, ethnicity, insurance status, and time in HIV care based on HIV clinic use.

### Recruitment

#### Aim 1

Clinics will identify a convenience sample of 5 staff members to participate in qualitative interviews and complete surveys at each of the 5 sites. The interviewed staff will include key staff such as the nursing supervisor, physicians, social workers, and administrators. At the conclusion of the interviews, staff participants will be sent a Qualtrics Software (Provo) survey to complete. Additionally, 5 patients, identified by key staff for their experience with MH and SUD, from each clinic will be recruited to complete an anonymous survey through Qualtrics.

#### Aim 2

We will work with each of the clinics to identify 3 patients and 5 key staff members in years 2, 3, and 5 of the study for individual interviews (45 patients and 75 key staff participants will be selected using purposive sampling with maximum variation to ensure diversity in perspectives. We expect representation of persons living with HIV of different races, ages, duration of engagement in HIV clinical care, education level, socioeconomic status, and insurance status. Unique patients and staff will be selected at each time point. Recruitment for each participant group will take place at each time point (years 1-4) until thematic saturation is reached.

#### Aim 3

Patients will be recruited to +STEP by their clinic staff. Participating patients will complete ePROs at least once per year. Their ePRO records and historical clinical data will be used for both process and clinical outcomes. The target sample size for is 4000 participants across sites, which would allow us to detect for an odds ratio of 1.29.

### Data Sources

For aim 1 of this study, we will collect data using a patient telephone survey, a staff survey, and qualitative interviews with key staff to understand current capacity and infrastructure to inform implementation. The patient survey measured the acceptability and accessibility of ePROs and telehealth services. The key staff survey included the organizational readiness to implement change instrument to determine perceived institutional readiness [38]. Interviews with key staff explored technological capacity and organizational readiness for change using the CFIR. Additional methodological details for aim 1 have been published in BMC Health Services Research [39]. The Implementation data will be abstracted from scheduling logs, training logs, and the electronic medical record (EMR) at each clinic site every 2 months to characterize the clinic and patient adoption and penetration of +STEP. We will supplement these reports with manual chart abstraction if needed to understand any gaps in the data. For aim 2, patients and staff who meet eligibility criteria will be invited to participate in an hour-long qualitative interview. All interviews will be designed to elucidate (1) perceptions of implementation strategies and (2) anticipated and observed barriers and facilitators to +STEP delivery. These data will be used to refine implementation over time. Research staff with expertise in qualitative research and program evaluation will lead in-depth interviews using standardized interview guides. Interview guides will elucidate contextual factors and will be informed by constructs from the 5 domains of CFIR, predominantly intervention characteristics and the inner setting domains, and the associated implementation strategies designed to target those factors. These factors may influence clinics’ ability to implement and sustain +STEP and ultimately improve outcomes. In addition, the interviews will evaluate the acceptability of +STEP, a key component of Proctor’s Implementation Framework [32]. For aim 3, clinical data will be extracted from the EMR, scheduling data, and training logs. Historical controls will be the percentage of MH and SUD diagnoses among patients and will be collected at the start of the study. We will extract ePROs, referrals for clinical encounters, visits attended and missed, and other HIV care continuum outcomes (eg, VL). We will quantify the proportion of participants with ePROs indicative of MH and SUD, and suboptimal ART adherence. We will quantify the number and proportion of clinical encounters scheduled and attended related to HIV, MH, substance use, social services, case management, and telemedicine visits.

### Outcomes

Targeted outcomes for this study are outlined in Table 1.

**Table 1:** Outcomes including units, evaluation metrics, data source, and timing for aim 1, 2, and 3.

Outcome, concept, and unit					Evaluation metrics		Data source		Timing
**Aim 1**									
	**Outcome: readiness, acceptability, and accessibility**								
		**Concept: organizational readiness**							
			**Unit: key staff**						
				Interviews to understand capacity and infrastructure		Interviews with key staff		Preimplementation	
				Survey of organizational readiness to implement change instrument		Key staff survey		Preimplementation	
		**Concept: acceptability and accessibility**							
			**Unit: patients**						
				Survey of acceptability and accessibility of ePROs^a^ and telehealth services		Patient survey		Preimplementation	
	**Outcome: fidelity**								
		**Concept: degree to which an intervention is delivered as intended**							
			**Unit: clinics**						
				Review of use of intervention and implementation strategies		Implementation team		Every 2 months	
			**Unit: patients**						
				Use of implementation strategies, consistency of ePROs, telemedicine uptake		EMR^b^ and ePRO data, scheduling data		Quarterly as part of reports	
	**Outcome: process**								
		**Concept: clinic penetration**							
			**Unit: clinics**						
				Percentage of staff taking CME^c^ training, percentage of ePROs delivered and percentage generating response, number of referrals to services, number of telemedicine referrals		Implementation team, EMR, ePRO scheduling data		Every 2 months	
		**Concept: patient penetration**							
			**Unit: patients**						
				Percentage of patients enrolled; percentage completing ≥1 ePRO report; average number of PROs completed per patient; number of MH^d^, SUD^e^, or telemedicine visits		EMR, ePRO, scheduling data		Quarterly as part of reports	
**Aim 2**									
	**Outcome: implementation**								
		**Concept: facilitators, barriers, and perception of implementation strategies**							
			**Unit: patients and staff**						
				Patient and staff perspective of implementation, as framed by CFIR^f^		Patient and staff interviews		Annually	
**Aim 3**									
	**Outcome: ePROs**								
		**Concept: MH and substance use**							
			**Unit: patients**						
				Percentage of patients reporting MH, alcohol and illicit drug use, including OUD^g^; low ART^h^ adherence		ePRO data		Every 6 months	
		**Concept: ART adherence**							
			**Unit: patients**						
				Association between ePRO, intervention, and ART adherence		ePRO data		Every 6 months	
	**Outcome: referrals for services**								
		**Concept: counseling, psychiatry, addiction medicine, and rehabilitation**							
			**Unit: patients**						
				Referrals to specialty care		Training logs, EMR and scheduling data		Every 6 months	
	**Outcome: use of services**								
		**Concept: counseling, social work, MH, and addiction services**							
			**Unit: patients**						
				Percentage of patients with clinical encounters with counselor, social work, MH, addiction services		Training logs, EMR and scheduling data		Every 6 months	
	**Outcome: telemedicine encounters**								
		**Concept: MH and substance use**							
			**Unit: patients**						
				Percentage of encounters completed through telemedicine		Training logs, EMR and scheduling data		Every 6 months	

^a^ePRO: electronic patient reported outcome.

^b^EMR: electronic medical record.

^c^CME: continuing medical education.

^d^MH: mental health.

^e^SUD: substance use disorders.

^f^CFIR: consolidated framework for implementation research.

^g^OUD: opioid use disorder.

^h^ART: antiretroviral treatment.

### Analysis Plan

**Figure 2:**
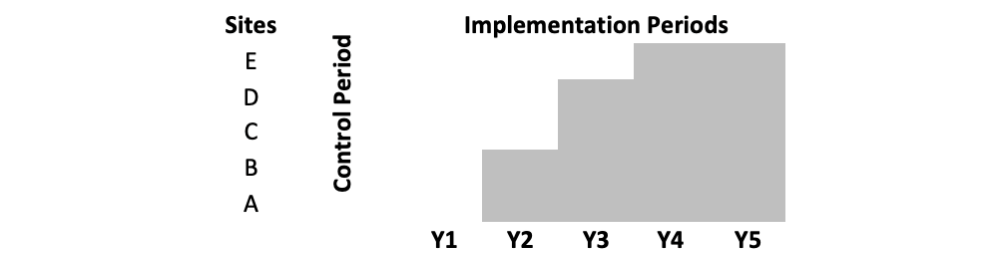
The stepped-wedge study design in which the intervention (patient-reported outcomes) is introduced at sites across 3 years of the study (Years 2-4). Y: years.

The analysis process will align with a stepped-wedge study design and each aim of the study (Figure 2). Aim 1 is to conduct readiness, acceptability, and accessibility assessment and implement +STEP. Data collected during the readiness, acceptability, and accessibility assessment will be analyzed using descriptive and frequency statistics for the staff and patient survey and by a rapid qualitative analysis of qualitative interviews with key staff members from the 5 participating sites. The team will use a data reduction analysis method by coding and summarizing each transcript by code before integrating the themes across all interviews [40]. This process will elucidate each clinic’s protocol for completing annual screening and reporting of MH and SUD, clinic procedures for psychosocial service provision, including on-site services and details of referral options in the community and beyond, and the use of telemedicine if applicable.

Aim 2 is focused on describing implementation strategies addressing barriers to uptake of +STEP in 5 RWHAP clinics using CFIR. The analytic strategy for aim 2 is framework-guided rapid qualitative analysis (F-RQA) of interview transcripts [40]. We will develop an interview guide informed by the domains and constructs of CFIR and using the recommended CFIR interview questions. In alignment with F-RQA, a preliminary summary table will be developed to correspond to the domains and constructs reflected in the interview guide. Once both processes are complete, the research team will independently extract relevant data from a single transcript, including pertinent quotes, and organize them in the draft summary table. Draft summary tables from these team members will be compared to resolve discordance. A modified summary table will then be used on a second interview before finalizing the summary table, which will be used for data analysis of all subsequent interview transcripts. This analytic process will provide an in-depth understanding of implementation as it is ongoing and will allow us the opportunity to share this information with clinics during quarterly meetings with participating clinics [41].

Aim 3 includes the measurement of the impact of implementing +STEP on diagnoses, referrals, and health care use related to MH, SUD, and HIV by comparing clinical outcomes from patients receiving these interventions (ePROs and telemedicine) with historical controls. This analytic process will begin with descriptive analyses to illustrate characteristics of patients, including age, race, gender, sex, and time in HIV care for each site and overall. Historical control data will be compared to the prospectively collected ePRO data and percentages. As it will be difficult to identify certain historical values, we will note any uncollectable data and present descriptive statistics for +STEP patients alone. For all other outcomes, we will perform generalized linear mixed effects models to account for the within-clinic correlation. These models will be performed with and without covariate adjustment to determine if any potential patient population changes (eg, more persons living with HIV newly engaged in care receiving +STEP) confound the effect of STEP+ on the identification of MH diagnoses, referral to services or actual service use. We will determine if random effects are necessary and if they can be ignored, we will use logistic regression models for each outcome. For each model, all assumptions will be verified, and goodness-of-fit will be assessed.

### Ethics Approval

This study received approval from the UAB’s institutional review board (300005613).

## Results

**Figure 3:**
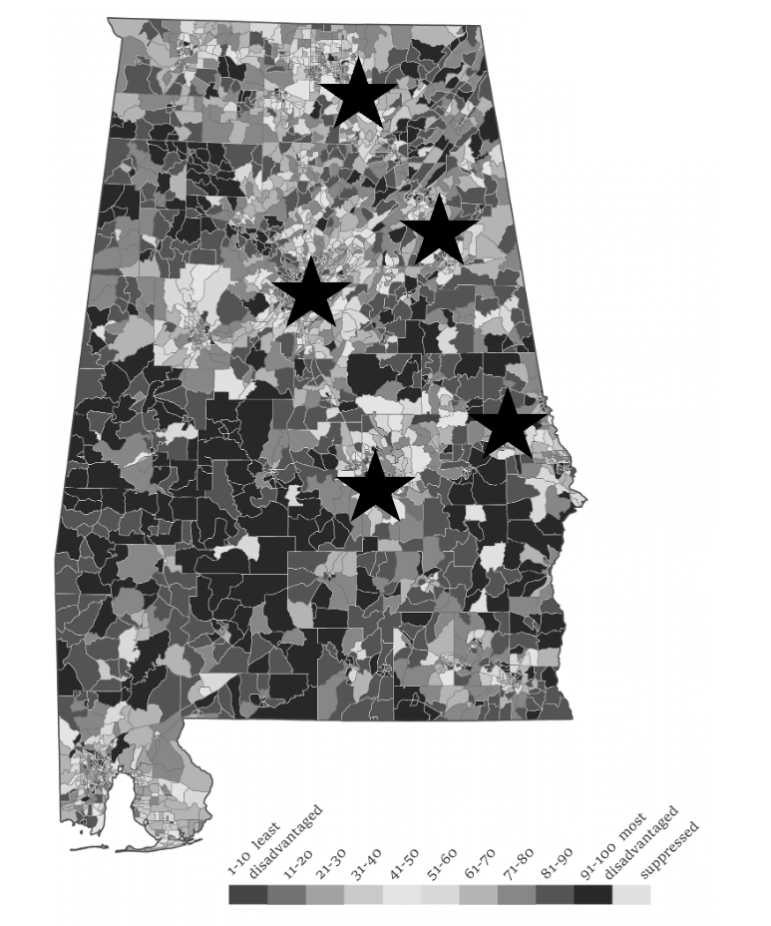
Stars indicate the location of RWHAP (Ryan White HIV/AIDS Program) clinics. Zip codes are shaded in accordance with the level of disadvantage based on the Area Deprivation Index, with White being the least disadvantaged and Black being the most disadvantaged.

In 2021, at the initiation of the study, the participating RWHAP-funded clinics provided care to a total of 4065 persons living with HIV (Table 2). The number of patients at each clinic ranged from 200 to 1819 (Table 2). These clinics are situated in the some of the most disadvantaged regions of Alabama based on the area deprivation index (Figure 3). The sociodemographic traits of persons living with HIV who are receiving care at RWHAP clinics in Alabama are as follows: median age of 47 years; 2622 (76%) cisgender men; 2273 (65%) non-Hispanic Black; 372 (10%) with unstable housing or homelessness. In 2019, there were 4065 persons living with HIV receiving care at one of the participating +STEP sites.

**Table 2:** Summary of sites participating in +STEP (N=4065).

Sites		Persons living with HIV, n (%)
**Ryan White Funded Clinics in Alabama^a^**		
	University of AL Family Clinic	300 (7.38)
	Thrive Federally Qualified Health Services Center	956 (23.52)
	Health Services Center	562 (13.83)
	Medical Advocacy and Outreach	1819 (44.75)
	Unity Wellness Center	428 (10.53)

^a^Data from 2020; 2021 data unavailable for this site.

Initial findings from aim 1 of this study are available at BMC Health Services Research [42]. Using data from patient surveys and key staff members’ qualitative interviews, this study found that all participating RWHAP-funded clinics were ready and willing to implement and integrate ePRO screening for MH and SUD followed by telehealth services as part of their treatment strategy. Patients reported comfort with technology, such as smartphones and computers, and telehealth; however, patients expressed concerns around not having a physical examination and less in-person interaction.

Future results of this study will be collected in alignment with the study timeline (Figure 1). As part of aim 2, we anticipate the results will be used to iteratively refine the formal implementation blueprint, which includes barriers and facilitators to +STEP delivery in routine care. Results will be instructive for the continued implementation of +STEP at RWHAP clinics and future sites. We anticipate that the results of aim 3 will demonstrate that +STEP will improve the diagnosis and treatment of MH and SUDs. We expect that +STEP will result in an increased number of referrals for evidence-based care of these conditions, an increased proportion of patients in treatment for both psychosocial comorbidities and HIV, and an increased quality of data used to monitor persons living with HIV in care at RWHAP clinics at the individual and clinic levels.

## Discussion

### Principal Findings

This will be the first study to evaluate the integrated implementation of 2 effective evidence-based technologies—ePROs and telemedicine—in serving people living with HIV in Alabama. We hypothesize that this study will provide foundational evidence demonstrating ePROs as an effective intervention to increase routine screening, referrals, and treatment for MH and SUDs. The results of this study will also include a refined blueprint for the implementation of ePROs at HIV clinics based on participant study–team meetings and evaluation of implementation over the course of the study.

### Comparison to Previous Work

A systematic review of ePRO randomized control trials identified consistent methodological limitations in the existing literature [14]. These limitations included lack of consensus on the structure of ePRO interventions, including how questionnaire results were provided to clinical providers, limited provider training on the interpretation of ePRO results, and a disconnect between ePROs and targeted outcomes, such as exploring the impact of ePROs on distal clinical outcomes in lieu of proximal process outcomes [14,43-45]. This study will address the existing limitations by including provider training on ePROs among persons living with HIV and the measurement of process outcomes to inform an implementation blueprint for ePROs to identify MH and SUDs in HIV clinics.

### Future Directions

The results of this study will provide evidence for future research expanding access to ePROs for improved diagnosis linked to telemedicine access to accelerate patients along the continuum of care from MH and SUD diagnosis to treatment. We will also use study findings to tailor future implementation research to the needs of African American communities, rural residents, historically marginalized communities, and those affected by intersectional stigmas, including gay men, transgender communities, and people who use drugs. These findings will have applicability in RWHAP-funded clinics in other parts of the Deep South and regions identified as priority sites for ending the HIV epidemic, which is a national initiative to scale up science-based strategies to diagnose, treat, prevent, and respond to HIV [46]. Implementing evidence-based services to support the mental and substance use needs of persons living with HIV is foundational to accelerating overall well-being and individual and population level viral load suppression.

### Limitations

This study is not without potential limitations. The initiation of this study took place during the COVID-19 pandemic, which affected community partners capacity to fully engage in the early stages of the study. However, COVID-19 has increased patients’ experience and comfort with telemedicine, aiding in the transition to ePROs and telemedicine for this study. Notably, during COVID-19, several clinics developed their own telemedicine programs out of necessity rather than waiting for the +STEP implementation. This means that many clinics are already providing MH services virtually, but few, if any, are using telehealth for addiction treatment services. Additionally, our community partners participate in others research studies and may have experienced study fatigue. Further, 1 site declined participation after the study was funded, citing COVID-19–related concerns and clinical capacity issues. Issues with clinic research saturation and COVID-19–related barriers will be monitored throughout the implementation of this study to try to mitigate overburdening our partners with research tasks when possible.

### Conclusions

Achieving the end of the HIV epidemic in the United States necessitates programs that accelerate movement across the MH and SUD care continuum from diagnosis to treatment for persons living with HIV. Scaling these services represents a path toward improved treatment outcomes with both individual health and population-level prevention benefits of sustained HIV viral suppression in the era of undetectable=untransmittable (U=U). This study will address this evidence gap through the evaluation of the implementation of +STEP to establish the necessary systems and processes to screen, identify, and better treat substance use and MH for people living with HIV.

## Abbreviations

ART: antiretroviral treatment
CFIR: consolidated framework for implementation research
EMR: electronic medical record
ePRO: electronic patient reported outcome
F-RQA: framework-guided rapid qualitative analysis
MH: mental health
PHQ-9: Patient Health Questionnaire-9
RWHAP: Ryan White HIV/AIDS Program
SUD: substance use disorders
U=U: undetectable=untransmittable
UAB: University of Alabama at Birmingham
VL: viral load

## Data Availability

Since this is an ongoing study, data are not yet publicly available, but may be available upon request to principal investigator EE at eeaton@uabmc.edu.
